# Natural Modulators of Endosomal Toll-Like Receptor-Mediated Psoriatic Skin Inflammation

**DOI:** 10.1155/2017/7807313

**Published:** 2017-08-13

**Authors:** Chao-Yang Lai, Yu-Wen Su, Kuo-I Lin, Li-Chung Hsu, Tsung-Hsien Chuang

**Affiliations:** ^1^Immunology Research Center, National Health Research Institutes, Miaoli 35053, Taiwan; ^2^Genomics Research Center, Academia Sinica, Taipei 115, Taiwan; ^3^Institute of Molecular Medicine, College of Medicine, National Taiwan University, Taipei 10002, Taiwan; ^4^Program in Environmental and Occupational Medicine, Kaohsiung Medical University, Kaohsiung 807, Taiwan

## Abstract

Psoriasis is a chronic inflammatory autoimmune disease that can be initiated by excessive activation of endosomal toll-like receptors (TLRs), particularly TLR7, TLR8, and TLR9. Therefore, inhibitors of endosomal TLR activation are being investigated for their ability to treat this disease. The currently approved biological drugs adalimumab, etanercept, infliximab, ustekinumab, ixekizumab, and secukizumab are antibodies against effector cytokines that participate in the initiation and development of psoriasis. Several immune modulatory oligonucleotides and small molecular weight compounds, including IMO-3100, IMO-8400, and CPG-52364, that block the interaction between endosomal TLRs and their ligands are under clinical investigation for their effectiveness in the treatment of psoriasis. In addition, several chemical compounds, including AS-2444697, PF-05387252, PF-05388169, PF-06650833, ML120B, and PHA-408, can inhibit TLR signaling. Although these compounds have demonstrated anti-inflammatory activity in animal models, their therapeutic potential for the treatment of psoriasis has not yet been tested. Recent studies demonstrated that natural compounds derived from plants, fungi, and bacteria, including mustard seed, *Antrodia cinnamomea* extract, curcumin, resveratrol, thiostrepton, azithromycin, and andrographolide, inhibited psoriasis-like inflammation induced by the TLR7 agonist imiquimod in animal models. These natural modulators employ different mechanisms to inhibit endosomal TLR activation and are administered via different routes. Therefore, they represent candidate psoriasis drugs and might lead to the development of new treatment options.

## 1. Introduction

Psoriasis is a common immune-mediated chronic inflammatory skin disease that affects the quality of life of 2%-3% of the global population. Psoriasis is typically associated with red, scaly, raised plaques resulting from a marked thickening of the epidermis induced by enhanced keratinocyte proliferation, leukocyte infiltrates in the epidermis and dermis, and inflammation [1–5]. Leukocyte infiltrates in psoriatic lesions primarily comprise dendritic cells (DCs), macrophages, neutrophils, and T cells. DCs generate multiple proinflammatory cytokines, including TNF-*α*, IL-1*β*, IL-6, and IL-23, that promote the development of psoriasis. TNF-*α* is a potent proinflammatory stimulus that promotes IL-23 production in DCs. IL-1*β* can activate IL-17 secretion from Th17 cells. IL-6 protects cutaneous T cells from Treg suppression and promotes Th17 participation in inflammation. Together, these immune cells and cytokines promote the inflammatory responses that underlie the development of psoriatic lesions.

Psoriasis can result from an interplay between genetic factors and external factors, including microbial infections, skin injuries, immune disorders, environmental influences, weather, and stress [6–15]. Nevertheless, the molecular mechanisms underlying the pathogenesis of this disease are not yet fully understood. TLRs are a family of pattern recognition receptors (PPRs) that localize to the cell surface or intracellular vesicles and are responsible for recognizing pathogen-associated molecular patterns (PAMPs) associated with microbes and danger-associated molecular patterns (DAMPs) released from dead cells in damaged tissues. A group of intracellular TLRs referred to as endosomal TLRs contributes to the pathogenesis and development of psoriasis by sensing endogenous DNA and RNA released from dead cells. In this review, we discuss current knowledge on the mechanism underlying endosomal TLR activation and the link between endosomal TLR activation and the pathogenesis of psoriasis. This mechanism can inform the development of therapeutics for psoriasis that target endosomal TLRs. Synthetic antagonists of endosomal TLRs are currently being developed. Natural products from plants, fungi, and bacteria are promising candidate drugs in this context because of their diverse structures and bioactivities. Many natural compounds have demonstrated acceptable safety profiles and immunomodulatory activity [16, 17]. We also discuss recently identified natural compounds that inhibit endosomal TLRs and reduce psoriatic inflammation via different mechanisms.

## 2. Toll-Like Receptors

The innate immune system is the first line of host defense to microbial infections. Innate immune cells use a diverse variety of PPRs including TLRs, nucleotide-binding oligomerization domain- (NOD-) like receptors (NLRs), C-type lectin-like receptors (CLRs), retinoic acid-inducible gene- (RIG-) I-like receptors (RLRs), and intracellular DNA sensor proteins to detect a wide variety of microbial PAMPs that initiate intermediate innate immune responses and lead to the development of adaptive immune responses [18–29]. Of them, TLRs are the most well-characterized PRRs. Thirteen TLRs have been identified in mammals, and ten of these (TLR1–10) are expressed in humans [30–35]. Human TLRs are strongly expressed in multiple types of immune cells, including DCs, macrophages, monocytes, natural killer cells, B cells, and T cells. They are also expressed in other cell types, including keratinocytes, chondrocytes, endothelial cells, and fibroblasts. Human TLRs are type I transmembrane receptors that feature an extracellular domain, a transmembrane region, and a highly conserved cytoplasmic region. The extracellular domain consists of multiple leucine-rich repeats (LRRs). The cytosolic region contains a Toll/interleukin-1 receptor (TIR) domain that mediates protein-protein interactions with the TIR domains of MyD88 adaptor protein family members, and these interactions initiate downstream intracellular signaling pathways [35–41].

**Figure 1:**
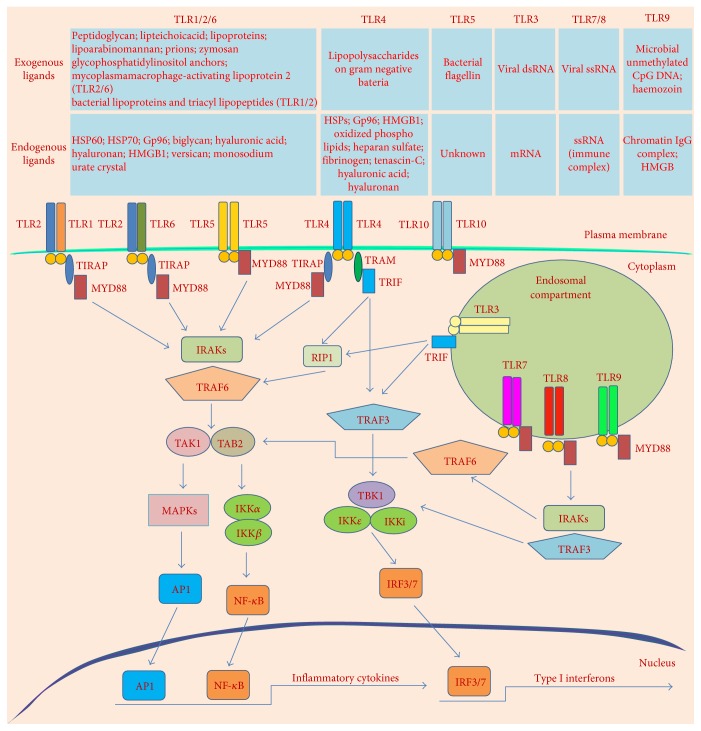
TLR ligands and signaling pathways. TLRs localize to the cell surface and to intracellular vesicles such as endosomes where they respond to their exogenous and endogenous ligands as shown. The TLRs utilize adaptor proteins of the MyD88 family, including MyD88, TRIF, TIRAP, and TRAM, to initiate downstream signaling pathways that induce the activation of various transcription factors, including NF-*κ*B, AP-1, and IRF3/7, and the production of inflammatory cytokines and type I interferons.

TLRs interact with a diverse variety of microbial PAMPs via their extracellular domain (Figure 1). TLR2 recognizes a broad range of microbial components, including peptidoglycan, lipoteichoic acids, lipoproteins, lipoarabinomannan, glycophosphatidylinositol anchors, prions, and zymosan [42–48]. TLR2 and TLR6 form a complex that selectively recognizes mycoplasma macrophage-activating lipopeptide 2, whereas a heterodimer composed of TLR2 and TLR1 selectively recognizes bacterial lipoproteins and triacyl lipopeptides. Natural ligands of TLR10 have not yet been identified [49–51]. TLR4 is the primary receptor responsible for recognizing lipopolysaccharides on the outer membrane of gram-negative bacteria, and TLR5 recognizes flagellin, a component of bacterial flagella [52, 53]. The binding of members of the TLR3, TLR7, TLR8, and TLR9 subfamilies to their ligands is mediated by the recognition of nucleic acid-derived structures. TLR3 recognizes double-stranded RNA (dsRNA) generated during viral replication in infected cells [54]. TLR7 and TLR8 recognize single-stranded RNA viruses such as vesicular stomatitis virus and the influenza virus [55, 56]. TLR9 is required for the response to microbial unmethylated CpG DNA [57, 58]. Most CpG sites in mammalian cells are methylated, whereas microbial CpG sites are typically unmethylated; therefore, unmethylated CpG DNA is a microbial PAMP [59, 60]. In addition, TLRs recognize a wide variety of DAMPs released from dead cells at inflammatory sites (Figure 1). DAMPs recognized by TLRs include cellular components and stress-induced gene products such as extracellular matrix components, extracellular proteins, intracellular proteins, and nucleic acids [61, 62]. TLR2 recognizes heat shock proteins (HSPs), Gp96, biglycan, hyaluronic acid, hyaluronan, high-mobility group box 1 (HMGB1), versican, and monosodium urate crystal [63–71]. TLR4 recognizes HSPs, Gp96, HMGB1, oxidized phospholipids, heparan sulfate, fibrinogen, fibronectin, tenascin-C, hyaluronic acid, and hyaluronan [64, 69–79]. TLR3, TLR7, TLR8, and TLR9 are activated by host RNA and host DNA from necrotic cells [80–83].

Upon activation by PAMPs or DAMPs, TLR monomers dimerize, and their cytosolic domains subsequently recruit adaptor proteins from the MyD88 family (MyD88, TRIF/TICAM-1, TIRAP/Mal, TIRP/TRAM, and SRAM), thereby initiating downstream signaling pathways [84] (Figure 1). With the exception of TLR3, which signals via a TRIF-dependent signaling pathway, all TLRs signal via a MyD88-dependent pathway. In the MyD88-dependent pathway, a MyD88/IRAK1/IRAK4/TRAF6 complex activates TAK1, thereby promoting the activation of several transcription factors, including NF-*κ*B and AP-1. TLR3 and TLR4 recruit TRIF to activate NF-*κ*B, AP-1, and IRF3/7. NF-*κ*B and AP-1 activation involves TRAF6 and RIP, and IRF3/7 activation is mediated by a TBK1-IKK*ε*/IKKi complex (Figure 1). These transcription factors are key regulators of the expression of adhesion and costimulatory molecules and the production of various inflammatory cytokines required for the maturation, differentiation, and proliferation of DCs, natural killer cells, and cytotoxic T cells [41, 85–88].

## 3. Endosomal Toll-Like Receptors

**Figure 2:**
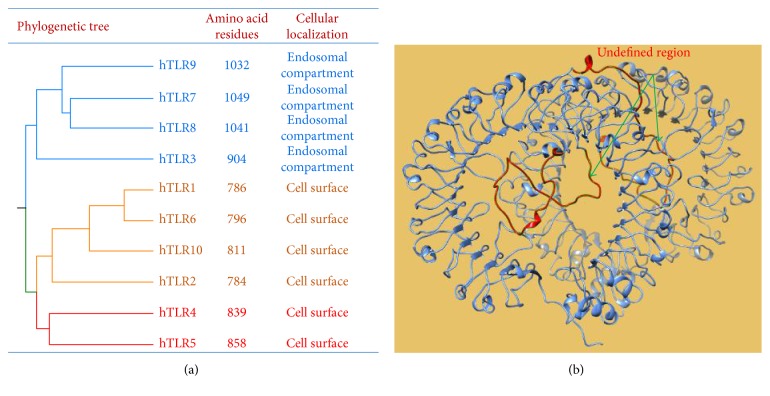
Subfamilies and the extracellular structure of human TLRs. (a) Human TLRs are divided into three phylogenetic subfamilies shown with different colors. The number and cellular location of amino acid residues are shown in the middle and right columns, respectively. (b) Computational modeling of the ectodomain structure of dimerized TLR7. Blue color shows the horseshoe-shaped solenoid structure of TLR ectodomain. Arrowheads indicate undefined regions (red color).

The ten human TLRs are divided into three phylogenetic subfamilies. The first subfamily comprises TLR1, TLR2, TLR6, and TLR10. The second subfamily comprises TLR4 and TLR5, and the third subfamily comprises TLR3, TLR7, TLR8, and TLR9 (Figure 2(a)). TLR1, TLR2, TLR4, TLR5, and TLR6 are expressed on the cell surface. In contrast, TLR3, TLR7, TLR8, and TLR9 localize to the endoplasmic reticulum and are trafficked to the endosomal/lysosomal compartment where they initiate cellular responses upon their activation by PAMPs and DAMPs. Therefore, these four TLRs are referred to as endosomal TLRs [89, 90]. In addition to their unique mechanism of ligand recognition, the four endosomal TLRs are also distinct from other TLRs with respect to protein length. Specifically, TLR7, TLR8, and TLR9 are composed of more than 1000 amino residues (Figure 2(a)), and their extracellular domain is longer than that of other TLRs. Most TLRs contains 19–25 LRRs arranged in a horseshoe-shaped solenoid structure that mediates ligand binding. TLR7, TLR8, and TLR9 have 25 LRRs and a unique undefined region/Z-loop between LRR14 and LRR15 (Figure 2(b)). Previous studies have shown that mouse and rat TLR8 has low activity levels and can only be activated by agonists in the presence of PolyT-ODN. The lack of a five-amino-acid motif in the undefined region/Z-loop is proposed to account for the weak activity of these two TLR8 homologues, suggesting that the undefined region/Z-loop plays a role in the activation of TLR7, TLR8, and TLR9 [91, 92].

Endosomal TLRs must be trafficked from the endoplasmic reticulum to specific cellular locations in order to be activated. The intracellular trafficking of endosomal TLRs is regulated by accessory proteins such as UNC-93 homolog B1 (UNC93B1) and specific adaptor proteins (APs). UNC93B1 directly interacts with endosomal TLRs in the endoplasmic reticulum, facilitates their transport to the Golgi apparatus via coat protein complex II (COPII) vesicles, and remains associated with them in endosomes. AP1 and AP2 are required for UNC93B1-mediated endosomal TLR trafficking. AP3 facilitates TLR9 trafficking from endosomes to lysosome-related organelles (LROs). In contrast to the effects of TLR9 activation at endosomes, TLR9 activation at LROs induces the production of type I interferons rather than proinflammatory cytokines [93–95].

After reaching the endosomal compartment, TLRs are cleaved by specific proteases, including asparagine endopeptidase (AEP) and cathepsins such as cathepsin B, cathepsin H, cathepsin K, cathepsin L, and cathepsin S [96–98]. For example, TLR9 is cleaved at its undefined region/Z-loop in endosomes, and this cleavage is a prerequisite for its activation. Interestingly, following proteolytic cleavage, the N-terminal and C-terminal portions remain associated with one another, and this association is required for protein function [98–102]. This raises the question of what the role of proteolytic cleavage in TLR activation is. Recent studies have shown that the cleaved and uncleaved receptors bind with equal affinity to their ligand [101, 103]. Nevertheless, ligand-induced TLR dimerization requires proteolytic cleavage to relieve steric hindrance at the undefined region/Z-loop. This finding is consistent with the observation that endosomal acidification is required for endosomal TLR activation, as an acidic pH is required for the activation of cathepsins and most endosomal and lysosomal proteases [99–101].

## 4. The Role of Endosomal Toll-Like Receptors in the Pathogenesis of Psoriasis

**Figure 3:**
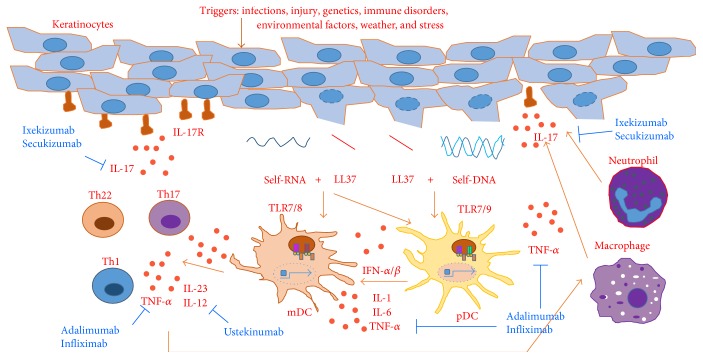
The role of endosomal TLRs in the development of psoriasis and the mechanism of action of biological drugs. Endosomal TLRs in plasmacytoid dendritic cells (pDCs) and myeloid dendritic cells (mDCs) can be triggered by self-DNA and self-RNA that forms complexes with LL37 upon its release from necrotic cells. Cytokines released by DCs further drive T cell-mediated inflammation by activating cytokines that promote leukocyte recruitment and keratinocyte activation and proliferation. Blue font: biological drugs inhibit effector cytokines in psoriatic inflammation.

In recent years, significant progress has been made in our understanding of the molecular mechanisms underlying the pathogenesis of psoriasis and the role of endosomal TLRs in this process. As shown in Figure 3, in the initiation phase, external triggers such as microbial infections and skin injuries induce the release of the antimicrobial peptide LL37 from keratinocytes and the release of self-DNA and self-RNA from dying cells to activate endosomal TLRs [104–106]. These TLRs can typically distinguish pathogen-derived nucleic acids from self-derived nucleic acids. Nucleic acids derived from viruses during cytosolic replication can be transported into endosomes during the process of autophagy where they activate endosomal TLRs. However, the localization of endosomal TLRs to intracellular compartments prevents their activation by self-nucleic acids under physiological conditions, because self-nucleic acids from dead cells in damaged tissues are unable to passively enter other cells and endosomes [107, 108]. Nevertheless, tolerance to self-nucleic acids can be evaded under some pathological conditions. For example, the antimicrobial peptide LL37 is upregulated and delivered to inflammatory sites in psoriatic skin where it forms complexes with self-nucleic acids to facilitate their entry into DCs and the subsequent activation of endosomal TLRs. These events render nonstimulatory self-nucleic acids into potent immune stimuli [104–106, 109, 110].

Endosomal TLRs are differentially expressed in different subsets of DCs. Plasmacytoid DCs (pDCs) express TLR7 and TLR9, and myeloid DCs (mDCs) express TLR7 and TLR8 [111]. Thus, LL37/RNA and LL37/DNA complexes can trigger the production of various proinflammatory cytokines, including TNF-*α*, IL-1, and IL-6, as well as type I interferons in pDCs, by activating TLR7 and TLR9. Cytokines produced by pDCs in turn promote the activation of mDCs. In addition, LL37/RNA complexes can directly activate mDCs via TLR7 and TLR8, thereby inducing the production of IL-12 and IL-23 in mDCs at psoriatic inflammatory sites (Figure 3). These cytokines activate T cells into Th1, Th22, and Th17 cells, thereby further activating cytokines that promote keratinocyte activation and proliferation and the recruitment of inflammatory cells such as neutrophils and macrophages to psoriatic lesions [112, 113]. Together, these events result in chronic cutaneous inflammation.

## 5. Evidence for the Involvement of Endosomal TLRs in Psoriatic Inflammation

The involvement of endosomal TLRs in the pathogenesis of psoriasis is supported by studies of imiquimod in mouse models of psoriasis. Imiquimod is a small molecular weight agonist of TLR7. Aldara™ is a 5% imiquimod cream approved for the treatment of genital warts and superficial basal cell carcinoma. In mouse models, consecutive topical application of Aldara cream to the ear or shaved back skin causes inflammation, and the responses to imiquimod in mice closely resemble symptoms of human psoriasis, including skin thickening and erythema. Aldara not only causes phenotypic changes consistent with psoriasis but it also induces leukocyte infiltration and activation of the IL-23/Th17 axis, suggesting that the mechanism of imiquimod-induced pathogenesis is similar to the pathogenesis of human psoriasis [114–116]. Consistent with these findings, there have been reports of psoriasis associated with the clinical application of imiquimod in patients with basal cell carcinoma or actinic keratosis with or without a history of psoriasis [116–118].

Direct evidence that endosomal TLRs are potential therapeutic targets of psoriasis treatment stems from clinical investigation of TLR antagonists. In a phase 2 clinical trial in patients with moderate to severe psoriasis, immune modulatory oligonucleotide- (IMO-) 3100, an antagonist of TLR7 and TLR9, was associated with a reduction in Psoriasis Area Severity Index (PASI) score. In an animal model of psoriasis established by intradermal injection of IL-23 in the dorsum, IMO-3100 inhibited epidermal hyperplasia. IL-23 injection altered the expression of more than 5000 genes and upregulated the expression of genes associated with IL-17 signaling [119]. Treatment with IMO-3100 modulated the expression of 1900 of genes and downregulated the expression of IL-17-regulated genes. IMO-8400 is a second generation IMO that antagonizes TLR7, TLR8, and TLR9. Similar to IMO-3100, IMO-8400 inhibited symptoms of psoriasis; however, IMO-8400 had a broader effect on the expression of IL-23-induced genes. In a phase 2a clinical trial evaluating the safety and efficacy of IMO-8400 compared with placebo in patients with moderate to severe plaque psoriasis, IMO-8400 did not cause any serious or severe adverse effects and it demonstrated clinical improvements. PASI-50 with IMO-8400 was 38% compared with 1% with placebo, and PASI-75 and PASI-90 with IMO-8400 were 17% and 2%, respectively, compared with 0% with placebo [119, 120]. These findings support the hypothesis that blocking endosomal TLR activation is a promising therapeutic approach for the treatment of psoriasis.

## 6. Strategies for Blocking Endosomal TLR-Mediated Inflammation

The design of strategies to block inflammatory responses elicited by the activation of distinct endosomal TLRs can be based on their unique functions and signaling mechanisms. These strategies are (1) neutralizing cytokines that mediate the effects of endosomal TLRs, (2) blocking TLR ligand interactions using TLR antagonists, (3) blocking TLR ligand interactions by sequestering TLR ligands, (4) blocking TLR activation by inhibiting proteasomal activity and endosomal acidification, (5) downregulating TLRs and their downstream signaling molecules, and (6) inhibiting signal transduction downstream of endosomal TLR activation. The feasibility of these strategies to treat psoriasis and other autoimmune diseases, including rheumatoid arthritis (RA) and systemic lupus erythematosus (SLE), has been demonstrated by the action mechanisms of the biological drugs and synthetic compounds (shown in Figure 3 and Table 1) that are currently being used or investigated.

### 6.1. Biological Drugs That Neutralize Effector Cytokines

Several biological drugs targeting cytokines that can be directly or indirectly generated by the activation of endosomal TLRs have been approved by FDA for the treatment of psoriasis. These biological drugs can be divided into three classes: TNF-*α* antagonists, IL-12/IL-23 inhibitors, and IL-17A inhibitors. The TNF-*α* antagonists adalimumab, etanercept, and infliximab have demonstrated strong efficacy in the treatment of moderate to severe psoriasis. Adalimumab is a fully humanized monoclonal antibody [121–123]. Etanercept is a recombinant fusion protein containing a TNF-*α* receptor ligand-binding domain and a human IgG Fc domain [124–126]. Infliximab is a chimeric monoclonal antibody [127–129]. TNF-*α* antagonists reduce inflammatory responses at psoriatic sites and downregulate the differentiation and function of Th17 cells. These findings suggest that TNF-*α* functions upstream of the IL-23/Th17 axis. Ustekinumab is a member of the class of psoriasis drugs that target IL-12/IL-23. It is a humanized monoclonal antibody that neutralizes the p40 subunit common to both IL-12 and IL-23, thereby preventing the binding of these cytokines to their receptors and the subsequent initiation of Th1- and Th17-mediated signaling pathways [130, 131]. Ustekinumab demonstrated a superior clinical effect to etanercept, suggesting that IL-23 plays a key role in the pathogenesis of psoriasis [132, 133]. The human monoclonal antibodies ixekizumab and secukizumab are members of the class of psoriasis drugs that target IL-17A. Multiple studies have shown that ixekizumab and secukizumab inhibit the expression of a wide variety of genes associated with Th17- and Th1-mediated inflammatory responses. Although these biological drugs have demonstrated efficacy in the treatment of psoriasis, their use is limited by their high cost and the fact that they must be administered by injection. Therefore, synthetic chemical drugs and natural inhibitors continue to be investigated and developed.

### 6.2. TLR Antagonists

IMO-3100 and IMO-8400 (discussed in Section 5) bind to endosomal TLRs, thereby preventing the interaction of endosomal TLRs with their agonists. Other immune inhibitory oligonucleotides that directly interact with TLR7 and TLR9, including IRS-954, DV117, and INH-ODN-24888, have also been developed [134–137]. The binding of these compounds to endosomal TLRs blocks their access to the agonists that trigger their activation. Although their therapeutic potential for the treatment of psoriasis has not yet been evaluated, these compounds have demonstrated immune inhibitory effects in preclinical and clinical SLE trials. CPG-52364 is another endosomal TLR inhibitor that blocks ligand-induced activation of TLR7, TLR8, and TLR9. Structurally distinct from other inhibitory oligonucleotides, CPG-52364 is a derivative of the small molecular weight chemical compound quinazoline. CPG-52364 has been reported to be well tolerated in clinical trials evaluating its effect in the treatment of several inflammatory autoimmune diseases, including psoriasis, RA, and SLE [137–139].

### 6.3. Compounds That Inhibit Endosomal Acidification, Proteasomal Activity, and Sequestering TLR Ligands

The antimalarial drugs chloroquine, hydroxychloroquine, and quinacrine are derivatives of quinine, a natural alkaloid isolated from the South American cinchona bark tree. Although they are primarily used to treat malaria, these drugs have long been used for treating skin diseases and reducing inflammation in RA and SLE. More recent studies demonstrated that these compounds function as inhibitors of TLR7, TLR8, and TLR9 [140, 141]. They are weak bases; therefore, their ability to inhibit endosomal TLR activation has been attributed to their ability to inhibit endosomal acidification. These antimalarial compounds have also been shown to directly interact with nucleic acid-based TLR ligands, thereby sequestering these ligands and preventing them from binding to endosomal TLRs. Bortezomib (Velcade) is one example of a drug that targets proteasomal activity. Bortezomib is a proteasome inhibitor approved for the treatment of multiple myeloma, and it has demonstrated inhibitory effects in several autoimmune disorders, including psoriasis, RA, and SLE, in animal models [142–144]. In addition, bortezomib has been shown to suppress the trafficking of TLR9 to endolysosomes, inhibit TLR9 activation, and reduce lupus- and psoriasis-associated inflammation [145, 146].

### 6.4. Compounds That Downregulate Endosomal TLRs and Inhibit TLR Signaling

Other small molecular weight chemical compounds that inhibit TLR signaling and TLR-mediated inflammatory responses in autoimmune diseases include SM934, ST-2825, IRAK4 inhibitors, and IKK2 inhibitors. SM934 (*β*-aminoarteether maleate), a derivative of artemisinin, possesses potent antiproliferative and anti-inflammatory properties. In a preclinical study, SM934 provided a significant protective effect in a mouse model of SLE. SM934 inhibits TLR activation by promoting the downregulation of TLR7, TLR9, and MyD88 mRNA expression and the inhibition of NF-*κ*B phosphorylation [147–149]. Other compounds that block NF-*κ*B activation and cytokine production by targeting molecules associated with TLR signaling have also been developed. For example, the peptide mimetic ST-2825 targets MyD88. ST-2825 interferes with MyD88-mediated recruitment of IRAK1 and IRAK4 to the TLR signalsome, thereby inhibiting TLR-mediated inflammatory responses. This compound has been shown to inhibit TLR9 activation and block the production of autoantibodies in B cells in SLE patients [150, 151]. AS-2444697, PF-05387252, PF-05388169, and PF-06650833 target the kinase activity of IRAK4 and have been investigated in preclinical or clinical studies for the treatment of multiple inflammatory and autoimmune diseases, including gout, sepsis, AR, and SLE [152, 153]. IKK2 is a subunit of I*κ*B kinase, a protein that controls NF-*κ*B activation and the production of TLR-induced inflammatory cytokines. The chemical compounds ML120B and PHA-408 inhibit IKK2 kinase activity and have exhibited anti-inflammatory effects in an animal model of arthritis [154–156].

## 7. Natural Inhibitors of Endosomal TLR-Mediated Psoriatic Inflammation

Many natural compounds derived from bacteria, fungi, and plants have long been known to exhibit immunomodulatory activity and have been used for the treatment of inflammation-related disorders [16, 17]. For example, retinoids, vitamin D, and their corresponding analogs are used in topical treatments for psoriasis [157, 158]. These compounds bind to their endogenous cellular receptors, thereby initiating the transcription of genes that suppress inflammation and inhibit cell proliferation. In addition, the following plant extracts and natural compounds have been reported to inhibit endosomal TLR-mediated psoriatic inflammation in animal models via different mechanism of actions shown in Table 1. These natural products have therapeutic potential in the treatment of psoriasis.

### 7.1. Mustard Seed

Mustard seed from mustard plant is a popular food seasoning worldwide, especially in Japan, India, and China. Mustard seed possesses several biological effects, including anti-inflammatory, antioxidant, and antitumor effects. These effects are mediated by multiple active components, including erucic acid, isothiocyanate, phenols, and phytin [159]. In mice, a diet supplemented with 5% mustard seed for three weeks attenuated imiquimod-induced psoriasis-like inflammation. Mustard seed inhibited the infiltration of various types of leukocytes, including DCs, macrophages, and T cells, into psoriatic lesions. In addition, PASI score significantly decreased in mustard seed-fed mice compared with control mice. Furthermore, NF-*κ*B, IFN-*α*, IL-17, and IL-22 levels decreased in the psoriatic lesions of mustard seed-fed mice compared with control mice [160]. However, the specific component mediating this effect and its molecular target in this context remains unclear.

#### 7.1.1. *Antrodia cinnamomea* (*A. cinnamomea)* Extract

*A. cinnamomea* is a species of fungus commonly used in Asia as a medicinal herb. It possesses a broad range of biological effects, including anti-inflammatory, antioxidant, antifatigue, and antitumor effects [161–163]. A previous study evaluated the effects of orally administered lyophilized extract from the fruit body of *A. cinnamomea* on disease severity in an animal model of imiquimod-induced psoriasis. *A. cinnamomea* extract reduced psoriasis-like inflammation, infiltration of CD4+ T cells, CD8+ T cells, and neutrophils, and the expression of TNF-*α*, IL-17A, and IL-22 in imiquimod-induced psoriatic skin lesions [164]. Although these observations support a role for *A. cinnamomea* in the treatment of endosomal TLR-mediated psoriasis inflammation, the effective component mediating these effects and the molecular target of *A. cinnamomea* in this context remain unclear.

### 7.2. Curcumin

Curcumin is a bright yellow powder obtained from the rhizome of several types of ginger plants. Chemically, curcumin is a diarylheptanoid belonging to the curcuminoid group. It has diverse bioactive effects, including anti-inflammatory, antioxidant, antitumor, and antiatherosclerotic effects [165–167]. Topical use of a curcumin-formulated gel has been shown to inhibit imiquimod-induced psoriasis-like inflammation. Curcumin treatment significantly inhibited imiquimod-induced epidermal hyperplasia and TNF-*α*, IL-1*β*, IL-6, IL17A, IL-17F, and IL-22 production in psoriatic lesions [168]. Curcumin is known to inhibit NF-*κ*B activation by inhibiting I*κ*B phosphorylation and degradation. NF-*κ*B signaling mediates the production of the inflammatory cytokines TNF-*α*, IL-1*β*, IL-6, and IL-23, which cooperatively induce the production of IL-17 cytokines and IL-22 from dermal *γδ* T cells. Therefore, curcumin might inhibit endosomal TLR-induced psoriatic inflammation by targeting NF-*κ*B signaling. Consistent with this hypothesis, curcumin-mediated inhibition of NF-*κ*B activation resulted in the downregulation of IL-17 and IL-22.

### 7.3. Resveratrol

Resveratrol is a stilbenoid, which is a type of natural phenol found in grapes, berries, and nuts. It possesses anti-inflammatory and antioxidant effects and is used as a dietary supplement [169, 170]. Oral administration of resveratrol diminished the severity of imiquimod-induced psoriasis-like inflammation in an animal model, and microarray analysis revealed that resveratrol treatment inhibited imiquimod-induced expression of IL-17A, IL-19, and IL-23p19. Resveratrol has been shown to inhibit LPS- and TNF-*α*-induced NF-*κ*B activation [171]. Thus, resveratrol might inhibit imiquimod-induced psoriatic inflammation by directly or indirectly targeting signaling of NF-*κ*B.

### 7.4. Thiostrepton

Thiostrepton, an antibiotic derived from several strains of *Streptomycetes*, functions as an antagonist of TLR7, TLR8, and TLR9. It was identified using a connectivity map screen for functional analogs of bortezomib. Subsequent studies demonstrated that thiostrepton inhibited TLR7-, TLR8-, and TLR9-mediated NF-*κ*B activation in a cell-based assay. It also reduced the production of TNF-*α* and IL-12/23p40 induced by R848 and LL37/RNA complexes (TLR7 and TLR8 ligands) and by CpG-ODN and LL37/DNA complexes (TLR9 ligands) in DCs. This natural antibiotic inhibited imiquimod-induced psoriasis-like inflammation in mice. Specifically, thiostrepton inhibited the accumulation of monocytes and DCs and the expression of TNF-*α*, IL-1*β*, and IL-8 in inflammatory lesions. Thiostrepton uses two mechanisms to block endosomal TLR activation. One mechanism depends on its proteasomal inhibitory activity, similar to the mechanism underlying bortezomib-mediated inhibition of endosomal TLR activation. The other mechanism depends on its ability to inhibit endosomal acidification [172].

### 7.5. Azithromycin

Similar to thiostrepton, azithromycin is an antibiotic isolated from *Streptomycetes*. Azithromycin possesses anti-inflammatory and immunomodulatory properties and is used to treat bacterial infections [173, 174]. Azithromycin inhibited imiquimod-induced expression of costimulatory molecules (CD40 and CD80) and cytokines (TNF-*α*, IL-10, IL-12p40, IL-12p70, and IL-23p19) in bone marrow-derived DCs (BMDCs), and topical treatment with azithromycin attenuated the severity of imiquimod-induced skin inflammation in an animal model of psoriasis. Azithromycin treatment inhibited keratinocyte hyperproliferation and the accumulation of DCs, CD4+ T cells, and CD8+ T cells in psoriatic lesions. The functional mechanism of azithromycin in this context is similar to that of thiostrepton. Azithromycin inhibited lysosomal acidification and the proteolytic processing of TLR7, thereby blocking imiquimod-induced NF-*κ*B and IRF7 activation in DCs [175]. In addition, a clinical study demonstrated that long-term oral azithromycin treatment improved PASI score in patients with chronic plaque psoriasis, further confirming the antipsoriatic function of this antibiotic [176].

### 7.6. Andrographolide

Andrographolide is a labdane diterpenoid isolated from the stem and leaves of *Andrographis paniculata*. This natural compound possesses anti-inflammatory activity and is currently used as a prescription medicine in China for the treatment of laryngitis, diarrhea, and RA. Intragastric administration of andrographolide alleviated imiquimod-induced psoriasis, but not IL-23-induced psoriasis, in mice. The therapeutic effect was dose-dependent, and treatment with 10 mg/kg andrographolide was as effective as treatment with 10 mg/kg etanercept in improving clinical scores in mice with imiquimod-induced psoriasis. Andrographolide inhibited imiquimod-induced expression of the genes encoding CD80, CD86, IL-1*β*, IL-6, and IL-23 in BMDCs. Treating BMDCs with andrographolide promoted MyD88 degradation and blocked the recruitment of TRAF6 to form signalsomes. Inhibiting autophagic proteolysis in BMDCs using NH_4_Cl or deleting the gene encoding microtubule-associated protein 1 light chain 3 (MAP1LC3B) abolished andrographolide-induced MyD88 degradation [177]. These findings suggested that andrographolide induces autophagic proteolysis of MyD88, thereby reducing psoriatic inflammation by inhibiting TLR-mediated cytokine production.

## 8. Conclusion

As summarized in Table 2, the studies described in this review indicate that natural products from plants, fungi, and bacteria inhibit the activation of endosomal TLRs via mechanisms that block their functions in the initiation and development of psoriasis. Compared with biological drugs, these natural modulators can be more cost-effective and are administered via different routes. Therefore, they are promising candidate drugs for the treatment of psoriasis, and they might inform the development of multiple treatment options.

## Figures and Tables

**Table 1:** Inhibitors for endosomal TLR-mediated inflammation.

Inhibitor	Natural/synthetic	Target and mechanism of action
IMO-3100	Synthetic	Antagonist of TLRs
IMO-8400	Synthetic	Antagonist of TLRs
IRS-954	Synthetic	Antagonist of TLRs
DV117	Synthetic	Antagonist of TLRs
INH-ODN-24888	Synthetic	Antagonist of TLRs
CPG-52364	Synthetic	Antagonist of TLRs
Chloroquine	Synthetic (quinine derivative)	Inhibits endosomal acidification or sequester TLR ligands
Hydrochloroquin	Synthetic (quinine derivative)	Inhibits endosomal acidification or sequester TLR ligands
Quinacrine	Synthetic (quinine derivative)	Inhibits endosomal acidification or sequester TLR ligands
Bortezomib	Synthetic	Inhibits TLR trafficking
SM934	Synthetic	Promotes downregulation of TLRs
ST-2825	Synthetic	MyD88 inhibitor
AS-2444697	Synthetic	IRAK4 inhibitor
PF-05387252	Synthetic	IRAK4 inhibitor
PF-05388169	Synthetic	IRAK4 inhibitor
PF-06650833	Synthetic	IRAK4 inhibitor
ML120B	Synthetic	IKK2 inhibitor
PHA-408	Synthetic	IKK2 inhibitor
Mustard seed	Natural (mustard plant product)	Inhibit NF-*κ*B activation and cytokine expression
*Antrodia cinnamomea* extract	Natural (*A. cinnamomea* product)	Inhibit cytokine expression
Curcumin	Natural (grapes product)	Inhibit NF-*κ*B activation and cytokine expression
Resveratrol	Natural (grapes product)	Inhibit NF-*κ*B activation and cytokine expression
Thiostrepton	Natural (*Streptomyces* product)	Inhibits endosomal acidification and proteasomal activity
Azithromycin	Natural (*Streptomyces* product)	Inhibits endosomal acidification and proteasomal activity
Andrographolide	Natural (*A. paniculata* product)	Promotes downregulation of myD88

**Table 2:** Key points of natural modulator in inhibition of psoriatic skin inflammation.

(1) Less cost
(2) More choice of administration routes
(3) More diverse sources
(4) More diverse chemical structures
(5) More diverse targets in endosomal TLR-mediated inflammatory signaling pathways
(6) More diverse mechanisms to block endosomal TLR-mediated inflammation
